# Management of anterior triangle swellings in a tertiary vascular centre with emphasis on the roles of duplex ultrasound, computed tomography angiogram and magnetic resonance angiogram: a case series

**DOI:** 10.1186/1757-1626-2-9112

**Published:** 2009-11-30

**Authors:** Gabrielle C Colleran, Kevin C Cronin, Ann M Browne, Niamh Hynes, Sherif Sultan

**Affiliations:** 1 Department of Surgery, NUI Galway, Galway University Hospital, Newcastle, Ireland; 2 Department of Radiology, NUI Galway, Galway University Hospital, Newcastle, Ireland

## Abstract

**Background:**

Anterior triangle masses pose an important clinical dilemma. It is very difficult to distinguish the potential pathologies pre operatively by clinical and radiological assessment.

**Case report:**

The first case highlights the management of a bilateral chemodectoma, the second case is a presentation of castleman's disease and the third is that of metastatic tonsillar adenocarcinoma. All three cases had a similar presentation and radiological appearance pre-operatively.

**Conclusion:**

Anterior triangle masses span the clinical spectrum of pathologies from chemodectoma to castleman's disease to carcinoma. Expert vascular and radiological management is required for optimum patient care and should take place in a tertiary referral centre. Duplex US, CTA and MRA are important pre operative assessment tools to ensure that adequate information regarding the relationship of the lesion to the carotid artery is available to the operating surgeon who should have vascular expertise as deliberate practice volume has been repeatedly shown to result in improved patient outcome.

## Introduction

Anterior triangle masses pose an important clinical dilemma as it is very difficult to distinguish the potential pathologies pre operatively by clinical and radiological means and we aim to illustrate this in the following case series. The anterior triangle is a complex operative field that requires significant anatomical knowledge and manual dexterity and should only be tackled by surgeons with extensive specialist experience in carotid and supra-aortic arch arterial intervention. Pre operative vascular assessment of the vascularity of an anterior neck lesion and its relationship to the carotid arteries to ensure optimum patient outcome as the following case series outlines.

## Case 1

A 55 year old lady admitted with bilateral neck swelling with an 8 year history of a lump on the left side of the neck and a 15 month history of the right sided lump. She had a past medical history of breast cancer in 1998.

On examination both lumps were palpable below the angle of the mandible and medial to it, they were mobile, not attached to the overlying skin, non tender and of normal colour and temperature. She had no evidence of cranial nerve involvement or endocrine abnormalities and no issue with blood pressure control.

**Figure 1:**
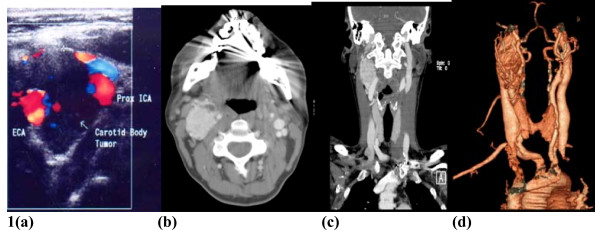
**A: Duplex US images of the neck demonstrating soft tissue masses between the left ICA and left ECA**. 1B, 1C, 1D: Axial CT post IV contrast and coronal 3D reconstruction showing right sided neck mass extending from the jugular foramen to the level of the mandible.

Carotid Duplex US showed a normal left common carotid artery but the left internal carotid and external carotid artery were splayed with evidence of a mass between the vessels, suspicious of a chemodectoma. The mass was 3 cm in diameter and appeared vascularised, fed by branches of the ECA. See Figure 1A.

The left mass was excised through a subadventitial dissection. The diagnosis of carotid body tumour was confirmed by histology, which revealed features consistent with a paraganglioma. Immunohistochemistry demonstrated that the mass was S100 positive, Ki67 low.

The right common carotid was normal but the right internal carotid artery and right external carotid artery were splayed by a soft tissue mass of 2.5 cm diameter. CT neck was carried out which revealed that the right sided mass extended superiorly to enlarge the jugular foramen and there was an intracranial component. See Figure 1B, 1C, 1D.

She had a full work-up with serological and urinalysis combined with a CT abdomen to rule out the presence of concomitant glomus tumours.

As CTA yielded inadequate visualization of the distal end of the CBT, MRI brain was performed which demonstrated a well defined enhancing lesion measuring 1.8 cm in diameter extending from localised posterolateral to the right carotid sheath and extending from the level of the carotid sheath up to the jugular foramen.

The right sided CBT was excised and postoperatively she developed a right vocal cord palsy and dysphagia particular for solids. This has resolved gradually.

## Case 2

**Figure 2:**
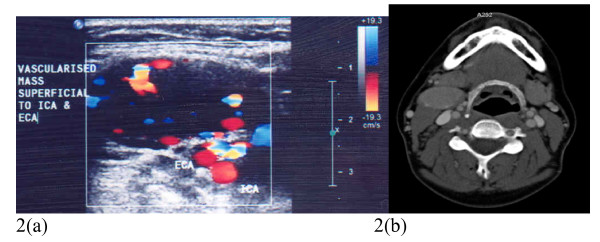
**2A: Carotid duplex image showing a highly vascular mass superficial to the ICA and ECA**. 2B: Axial CT of the neck post IV contrast showing a well circumscribed enhancing homogenous mass in the right parapharyngeal space.

A 36 year old female who presented with a 2 year history of intermittent flu like symptoms with accompanying neck swelling with no past medical history. A palpable neck mass was noted and a clinical diagnosis of a bronchial cyst was made. She was referred to an Endocrine Surgeon and a CT thorax was carried out which revealed a well circumscribed smooth enhancing homogenous mass in the right parapharyngeal space. It was lying anterior to the hyoid bone, carotid bifurcation and jugular vein and was compatible with but not typical of a carotid body tumour. See Figure 2B. At this point she was referred to the vascular service.

Carotid duplex imaging revealed a vascularised mass lying superficial to the right ICA and ECA which appeared to be fed by the ECA braches. See Figure 2A.

Following full work-up, the right sided lesion was completely excised and she had an uneventful operative course.

Histology of the mass revealed that it was a florid reactive follicular hyperplasia with increased nodal vascularity and interfollicular areas. The specimen was CD3 and CD20 positive with a diagnosis of Castleman's disease.

Due to this finding the patient was referred to the haematology services for the appropriate investigations including HIV, Toxoplasma, CMV and rheumatoid factor. Serology for Toxoplasma, CMV and hepatitis were all negative. CT TAP was normal with no mediastinal, para-aortic or pelvic adenopathy.

## Case 3

**Figure 3:**
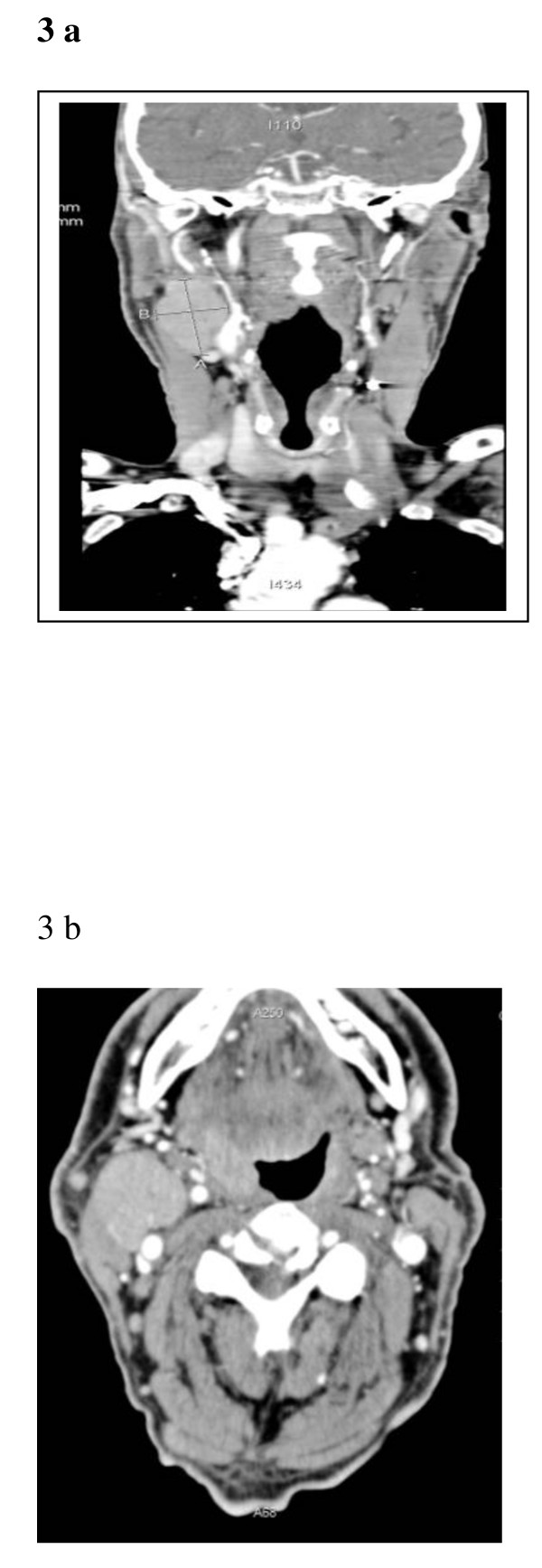
**A Coronal reconstruction of a CT neck post IV contrast demonstrating a right neck mass anterior to the hyoid b one and to the bifurcation of the CCA**. 3B: Axial CT neck image post IV contrast demonstrating a right sided neck mass anterior to the common carotid artery bifurcation.

A 77 year old male presented with a 1 cm right neck lump which grew to 2 cm over an eight month period before referral to the vascular service for assessment of his carotid arteries due to his history of having a reconstructed left carotid artery post trauma and intracranial haemorrhage. He had a past medical history of a 4 cm aortic aneurysm, gout, osteoporosis and osteoarthritis. His duplex USS revealed an occluded CCA and ICA with the appearance of an intraluminal collapsed prosthetic graft. A mildly vascularised irregularly shaped mass was identified medical to the right common carotid bifurcation. A differential diagnosis of a lesion compatible with carotid body tumour was made and pre-operative assessment was carried out. CT Thorax revealed a mass anterior to the hyoid bone and to the bifurcation of the common carotid artery into the internal carotid artery and external carotid artery, consistent with, but not diagnostic for carotid body tumour. See Figure 3A and 3B.

Complete Excision of the neck lesion was performed. Histology revealed that it was a squamous cell carcinoma with involvement of a lymph node. The most likely primary was from a head and neck carcinoma, most probably of nasopharyngeal or tonsillar origin. Oto-Rhino-Laryngology examination revealed a grossly abnormal tonsil which was removed in bulk with a diagnosis of right tonsillar carcinoma followed by a right radical neck dissection with post operative chemotherapy and radiotherapy. CT of the thorax and abdomen were negative for metastatic disease. Diagnosis was of a T2 N3 M0 carcinoma of the right tonsil.

## Discussion

Anterior triangle masses pose an important clinical dilemma as it is very difficult to distinguish the potential pathologies pre operatively by clinical and radiological means. All three of our patients presented with similar symptoms and clinical signs but had vastly different pathology. All had adequate resection by a specialist vascular surgeon and are doing well on follow-up.

Duplex US, CTA and MRA can all be used to assess the vascularity of a neck lesion. US can provide accurate information on the size, echogenicity, position and relationship to surrounding structures of a neck lesion, Duplex and colour flow US provide information regarding vascularity of the lesion [1]. Carotid body tumours (CBT), also known as chemodectomas are paragangliomas- vascular tumours of neural crest origin. CT or MRI are the diagnostic investigations of choice [2] although carotid body tumours are often first diagnosed on doppler ultrasonographic assessment. The typical appearance is of a splayed carotid bifurcation with anterior displacement of the external carotid artery (ECA) and posterior displacement of the internal carotid artery (ICA) and the internal jugular vein (IJV) [3]. In oncology patients with metastatic involvement of their cervical nodes CT and US have similar accuracy in detecting carotid artery invasion [4]. US can differentiate between low flow and high flow lesions but it is less able to define the boundaries of a lesion which is why MR is the best overall imaging tool due to its sensitivity for soft tissue contrast [5].

Pre operative vascular assessment of the vascularity of an anterior neck lesion and its relationship to the carotid artery is essential to ensure optimum patient outcome. As the three preceeding case reports demonstrate masses in the anterior triangle present with a very similar history and clinical examination which render definitive preoperative diagnosis difficult. However proximity to the carotid artery necessitates that adequate pre operative imaging of a mass in this region is carried out to determine its relationship to the carotid artery. However even in the absence of a definitive pre operative diagnosis of a relationship between the artery and the lesion, potential damage to the carotid artery can occur during resection. In carotid body surgery blood loss can be substantial and arterial reconstruction can be required in up to 25% of cases. The key to a successful outcome in Carotid body tumour surgery is to dissect in the subadventitial plane until the entire CBT is excised. The anatomic and technical details of operations for carotid body tumours are similar to those for carotid endarterectomy. Therefore it is essential that these procedures be carried out by highly specialized vascular surgeons. Deliberate practice volume has been repeatedly shown to improve outcome in carotid endarterectomy surgery and this can be extrapolated to the para-carotid surgical setting[6]. The consistency of improved outcomes in higher volume centres and in higher volume centres provides a strong argument for specialist vascular centres [7].

### Carotid Body Tumours

Surgical excision is the mainstay of treatment of CBTs with radiotherapy having an important palliative role and a central role in patients deemed unfit or unamenable to surgery [8].

**Castleman's disease **is a lymphoproliferative disorder associated with infections including Human Immunodeficiency Virus (HIV), Human Herpes Virus 8 (HHV-8) and cancers including Kaposi's sarcoma (KS), non-Hodgkin's lymphoma (NHL), POEMS syndrome and Hodgkin's Lymphoma. Treatment is surgical excision or chemotherapy, depending on staging. The consensus in the literature is that surgical excision is curative for patients with the monocentric subtype but surgical therapy is inappropriate in patients with multicentric disease and they should be candidates for multimodality therapy with combination chemotherapy. There are no reported recurrences after complete surgical excision in patients with unicentric castleman's disease [9].

Surgery and radiotherapy are the mainstays of treatment for tonsillar cancer with chemotherapy having an important adjuvant role in the management of patients with advanced disease such as the patient discussed in case 3. The presence of the SCC in the neck is a risk factor for inadequate margins this may be due to the proximity to the carotid and emphasises the importance of vascular involvement to ensure adequate resection is not compromised by proximity to the vessels [10]. Management of squamous cell cancer of the head and neck is very complex especially given the importance of maintenance of function of the mouth and pharynx for aesthetic and quality of life issues which are of paramount importance in treatment planning. Clinical trials have found little evidence to support combination chemotherapy over single agent regimens.

## Conclusion

Anterior triangle masses span the clinical spectrum of pathologies from chemodectoma to castleman's disease to carcinoma. All of these pathologies require differing long term management but as illustrated above are difficult to differentiate pre-operatively. Expert vascular and radiological management is required for optimum patient care and should take place in a vascular tertiary referral centre. Duplex US, CTA and MRA are important pre operative assessment tools to ensure that adequate information regarding the relationship of the lesion to the carotid artery is available to the operating surgeon who should have vascular expertise as deliberate practice volume has been repeatedly shown to result in improved patient outcome with decreased stroke and death rates.

## Consent

Despite our attempts, we were unable to obtain consent from the patients in this case report. All patients are anonimised and there is no reason to think that the patients or their families would object to publication.

## Competing interests

The authors declare that they have no competing interests to disclose at the time of submission.

## Authors' contributions

GC collected the patient history data, edited the text and wrote the paper. KC helped with data collection and pubmed literature search. AB chose the radiology images and provided descriptions of same. NH was involved in editing. SS was the overall supervisor and assisted with editing the manuscript. All authors approved and read the final manuscript.
